# Supplementary material for: Detection of dengue, malaria, and additional causes of acute febrile illness: The need for expanded testing, Bayelsa State, Nigeria
Source: PLoS Negl Trop Dis. 2025 Oct 10;19(10):e0013060. doi: 10.1371/journal.pntd.0013060 (PMC12533896; doi:10.1371/journal.pntd.0013060)
Supplement: Attachment — Submitted filename: Response to Reviewers.docx [file pntd.0013060.s003.docx]

Dear Editorial chief,

Thank you for the opportunity to publish with your prestigious journal, to the best of our ability we have reviewed and responded to your queries, instructions and suggestions. This is a rebuttal as requested of us.

Best regards

Rebuttal letter

**Reviewers' Comments:**

Reviewer's Responses to Questions

**Key Review Criteria Required for Acceptance?**

As you describe the new analyses required for acceptance, please consider the following:

**Methods:**

-Are the objectives of the study clearly articulated with a clear testable hypothesis stated?

-Is the study design appropriate to address the stated objectives?

-Is the population clearly described and appropriate for the hypothesis being tested?

-Is the sample size sufficient to ensure adequate power to address the hypothesis being tested?

-Were correct statistical analysis used to support conclusions?

-Are there concerns about ethical or regulatory requirements being met?

Reviewer #1: appropriate

Reviewer #2: 1. Is the Acro Biotech kit an Abbott dengue combo? The author should add a reference to Acro Biotech. Can that kit identify dengue primary or secondary infection? The reviewer thinks that the information is helpful for readers.

RESPONSE: No, Acro Biotech kit is not an Abbott dengue combo rapid test. This test is manufactured by Acro Biotech, USA.

I have added the reference in text as instructed. It can qualitatively identify both primary and secondary dengue by detection of NS1, IgM and IgG.

2. How much serum was used for RNA extraction, and how much ul of RNA was applied for real-time RT-PCR? The reviewer believes this information is helpful to PNTD readers.

Reviewer #3:

RESPONSE:

a. 140µl of plasma was used in the extraction
b. **15 μL** of RNA was used per PCR reaction. The PCR mix contained 14.0 µl of the respective master mixes, 1 µL of the IC and **15 µl of the sample**.

**Results:**

-Does the analysis presented match the analysis plan?

-Are the results clearly and completely presented?

-Are the figures (Tables, Images) of sufficient quality for clarity?

Reviewer #1: Yes

Reviewer #2: Out of 443 patients, 23 are qRT-PCR positive. Adding NS1, IgM, and IgG data for those 23 patients is better:

RESPONSE: yes, I have added the table to the manuscript, see table below as requested.

Reviewer #3: (No Response)

Table 2

| **PCR Positive patients (n=23)** | | | | |
| --- | --- | --- | --- | --- |
| **Patient ID** | **IgM** | **NS-1** | **IgG** | **DENV Serotype(s)** |
| 1 | - | - | Positive | 1, 4 |
| 2 | - | Positive | - | 3 |
| 3 | - | Positive | - | 3 |
| 4 | Positive | - | - | 3 |
| 5 | Positive | - | - | 3 |
| 6 | Positive | - | - | 3 |
| 7 | Positive | - | - | 3 |
| 8 | Positive | - | - | 3 |
| 9 | - | Positive | - | 3 |
| 10 | - | Positive | - | 3 |
| 11 | Positive | - | - | 3 |
| 12 | Positive | - | - | 3 |
| 13 | Positive | - | - | 3 |
| 14 | Positive | Positive | Positive | 3 |
| 15 | Positive | Positive | - | 3 |
| 16 | Positive | Positive | - | 2, 3 |
| 17 | Positive | Positive | - | 3 |
| 18 | Positive | Positive | - | 1 |
| 19 | Positive | - | - | 3 |
| 20 | Positive | - | - | 3 |
| 21 | Positive | - | - | 2 |
| 22 | Positive | - | - | 3 |
| 23 | Positive | - | - | 3 |

**Conclusions:**

-Are the conclusions supported by the data presented?

-Are the limitations of analysis clearly described?

-Do the authors discuss how these data can be helpful to advance our understanding of the topic under study?

-Is public health relevance addressed?

Reviewer #1: (No Response)

Reviewer #2: The discussion part is not enough. It should expand and include a discussion of each result.
RESPONSE: considering the word limit we initially limited our discussion to the key, most important findings. As instructed, we have revised to be more comprehensive and include next generation sequencing (NGS) methods and findings.

1. Why did the authors use the 31 years of age above and below as a seropositivity cut-off age in this study? Please explain it in the discussion part.

RESPONSE: In our analysis, we used 31 years as a cut-off age for DENV seropositivity based on the mean age distribution of our study population.

*2. Are there any differences in clinical symptoms between patients with dengue fever alone, malaria alone, and dengue fever and malaria complications? If so, this would be beneficial information for clinicians. Please explain in the discussion section.

RESPONSE: there were no observed significant differences in clinical signs between the groups of patients with malaria, dengue, or dengue – malaria coinfection.

*3. The positive rates for DENV, malaria, and mixed infections shown in Table 1 vary according to age distribution. Please discuss why this is the case in the discussion section.

RESPONSE: this has been done.

4. Of the 443 patients, 178 tested negative for DENV and malaria. In the discussion section, please discuss the possibility of other infectious diseases in these patients.

RESPONSE: Yes, this has been included in the results and discussion as suggested.

Reviewer #3: (No Response)

**Editorial and Data Presentation Modifications?**

Use this section for editorial suggestions as well as relatively minor modifications of existing data that would enhance clarity. If the only modifications needed are minor and/or editorial, you may wish to recommend “Minor Revision” or “Accept”.

Reviewer #1: No problem

Reviewer #2: (No Response)

Reviewer #3: (No Response)

**Summary and General Comments:**

Use this section to provide overall comments, discuss strengths/weaknesses of the study, novelty, significance, general execution and scholarship. You may also include additional comments for the author, including concerns about dual publication, research ethics, or publication ethics. If requesting major revision, please articulate the new experiments that are needed.

Reviewer #1: Akanbi et al. report on the prevalence of dengue virus infection in Yenagoa, Bayelsa State, Nigeria. They also investigated co-infection with dengue virus and malaria. Although Africa has been regarded as an endemic region for dengue virus, there are still relatively few studies on the subject. The authors collected specimens from patients with acute febrile illness in four hospitals in Yenagoa, and the study was well conducted. Additionally, they reported that dengue virus serotype 3 was the predominant type in the area during the study period in 2022. The authors suggest that some cases previously diagnosed as malaria may in fact have been dengue, which is an important observation.

Comments:
1. Table 1: The results for IgG-positive cases are missing, although this was mentioned in line 162.

RESPONSE: IgG results have been included in the table, modified as requested.

2.References 4 and 5: These refer to studies conducted in specific areas. It would be helpful to also cite a reference that discusses global dengue prevalence

RESPONSE: Reference has been added as instructed (Paz-Bailey et al 2024)

Lancet. 2024 Feb 17;403(10427):667-682. doi: 10.1016/S0140-6736(23)02576-X. Epub 2024 Jan 24.

3.Scientific writing: Some expressions are not appropriate for scientific reporting and should be revised. For example:

oLine 100: “(see Figure 1)” — consider rephrasing to integrate the figure reference more smoothly.

RESPONSE: This has been adjusted.

“As shown in Figure 1**”**

oLine 151: “we obtained ethical approval to …” — consider revising for a more formal tone.

RESPONSE: This has been adjusted. (Prior ethical approval was secured…)

Reviewer #2: The authors reported that 14.5% of febrile patients had acute dengue virus (DENV) infection, 42.4% had malaria, and 6.5% were coinfected with both in Bayelsa State, Nigeria, and that the findings highlight the need for routine DENV testing among patients with acute febrile illnesses in the region. The authors should consider the reviewers' comments. The author needs to write more clearly and accurately.

Reviewer #3: The manuscript PNTD-D-25-00583 submitted by Dayo Olufemi Akanbi is titled: The need for routine testing for dengue virus in Nigeria: detection and prevalence of dengue and coinfection with malaria among febrile patients in Yenagoa, Bayelsa State, Nigeria. Akanbi et al., used a cohort of febrile patients (N=443) collected in May-June 2022 from four hospitals to determine the seroprevalence of dengue infection, malaria and dengue/malaria co-infections. The authors performed rapid tests to detect DENV NS1, IgM and IgG, followed by multiplex DENV RT-PCR to serotype all NS1 and IgM positive samples.

They identified for the first time in Yenagoa (City in Bayelsa State) the presence of acute and past dengue infections with some patients experiencing malaria coinfection. This study provides evidence that emphasize the need to implement routine dengue surveillance in Nigeria.
The authors addressed an important question regarding the seroprevalence of flaviviruses, particularly dengue, in Nigeria, where data remain limited. The manuscript is concise and generally well written; however, minor revisions are recommended to improve the clarity of the manuscript.


The manuscript lacks references for the reagents used throughout the methodology section. The authors should include the sources and catalogue numbers where appropriate to ensure reproducibility.

RESPONSE: References have been added as instructed. The reagents used in this study were purchased through a third-party biotech agency known as Inqaba Biotech based in Ibadan, Nigeria.

L125-129: It is unclear whether the method used for plasmodium parasite screening is a standard or validated procedure. The authors should clarify this and provide an appropriate reference to support the methodology.

RESPONSE: Method used is standard and validated by the World Health Organization.

World Health Organization (WHO). Basic malaria microscope. Part 1 learner’s guide. 2010;180. The reference and for this WHO guide has been revised and cited in the body of the manuscript.

Molecular assay details: The molecular assay section requires further detail. The authors should provide comprehensive information regarding the primers used, the targeted gene, the reaction conditions, thermocycling parameters. Even a brief description will help ensuring reproducibility.

RESPONSE: Description has been included, furthermore, commercially available kits were used for extraction and detection, which didn’t require designing primer and probes, also, a brief description has been added, and references have also been added.

Statistical analysis: The manuscript does not clearly describe the statistical methods employed. The authors should specify the statistical tests used to claim associations/correlations between parameters.
RESPONSE: The specific statistical methods used in the study have been included in this section of manuscript.

Summary. L77: The word “signs” does not fit the narrative. Maybe the word “evidences” will be more appropriated.
RESPONSE: noted, and the text has been modified as suggested.

Title: The current title is long and may dilute the manuscript core findings. Consider shortening it to enhance clarity and focus. Suggested long title: Detection and Prevalence of Dengue and Coinfection with Malaria among Febrile Patients in Yenagoa, Bayelsa State, Nigeria. Suggested short title: Dengue Infection among Febrile Cases in Bayelsa State.

RESPONSE: Title has been modified to suit objectives and findings.

*Additional detail is needed regarding how rural and urban areas were defined in this study. Since spatial risk for vector-borne diseases may not align with the administrative definitions of rural and urban areas, clarifying the characteristics of rural and urban areas in this study is important.

RESPONSE: this also have been added to the latest version.

Figure 1: In the left panel of figure 1, it is unclear whether there is a between the Bayelsa state legend and the Nigeria boundary legend. If these are represented by different symbols or colors, the legend must be revised for clarity and visibility.

RESPONSE: The legends have been revised/recolored to clarify difference.

Figure 1. Colour of Map and legend have been revised as shown below:

*Study limitations and clinical data: The manuscript would benefit from a discussion of its limitations. Specifically, the absence of clinical data (e.g., WBC, liver enzyme levels, platelet counts etc…) limits interpretation of disease severity. Additionally, any analysis or discussion correlating clinical symptoms with coinfection status or dengue vs. malaria status would strengthen the manuscript.

RESPONSE: This has also been included.

[NOTE: If reviewer comments were submitted as an attachment file, they will be attached to this email and accessible via the submission site. Please log into your account, locate the manuscript record, and check for the action link "View Attachments". If this link does not appear, there are no attachment files.]

**Figure resubmission:**

While revising your submission, please upload your figure files to the Preflight Analysis and Conversion Engine (PACE) digital diagnostic tool, <https://pacev2.apexcovantage.com/>. PACE helps ensure that figures meet PLOS requirements. To use PACE, you must first register as a user. Registration is free. Then, login and navigate to the UPLOAD tab, where you will find detailed instructions on how to use the tool. If you encounter any issues or have any questions when using PACE, please email PLOS at [figures@plos.org](mailto:figures@plos.org). Please note that Supporting Information files do not need this step. If there are other versions of figure files still present in your submission file inventory at resubmission, please replace them with the PACE-processed versions.

**Reproducibility:**

To enhance the reproducibility of your results, we recommend that authors of applicable studies deposit laboratory protocols in [protocols.io](http://protocols.io/), where a protocol can be assigned its own identifier (DOI) such that it can be cited independently in the future. Additionally, PLOS ONE offers an option to publish peer-reviewed clinical study protocols. Read more information on sharing protocols at <https://plos.org/protocols?utm_medium=editorial-email&utm_source=authorletters&utm_campaign=protocols>
